# A High-Dimensional and Small-Sample Submersible Fault Detection Method Based on Feature Selection and Data Augmentation

**DOI:** 10.3390/s22010204

**Published:** 2021-12-29

**Authors:** Penghui Zhao, Qinghe Zheng, Zhongjun Ding, Yi Zhang, Hongjun Wang, Yang Yang

**Affiliations:** 1 School of Information Science and Engineering, Shandong University, Qingdao 266237, China; 201812603@mail.sdu.edu.cn (P.Z.); 201820314@mail.sdu.edu.cn (Q.Z.); 2 China National Deep Sea Center, Qingdao 266237, China; dzj@ndsc.org.cn (Z.D.); zy592@ndsc.org.cn (Y.Z.); 3 Public (Innovation) Experimental Teaching Center, Shandong University, Qingdao 266237, China

**Keywords:** fault detection, feature selection, data augmentation, high-dimensional sensor data, limited fault event, manned submersible

## Abstract

The fault detection of manned submersibles plays a very important role in protecting the safety of submersible equipment and personnel. However, the diving sensor data is scarce and high-dimensional, so this paper proposes a submersible fault detection method, which is made up of feature selection module based on hierarchical clustering and Autoencoder (AE), the improved Deep Convolutional Generative Adversarial Networks (DCGAN)-based data augmentation module and fault detection module using Convolutional Neural Network (CNN) with LeNet-5 structure. First, feature selection is developed to select the features that have a strong correlation with failure event. Second, data augmentation model is conducted to generate sufficient data for training the CNN model, including rough data generation and data refiners. Finally, a fault detection framework with LeNet-5 is trained and fine-tuned by synthetic data, and tested using real data. Experiment results based on sensor data from submersible hydraulic system demonstrate that our proposed method can successfully detect the fault samples. The detection accuracy of proposed method can reach 97% and our method significantly outperforms other classic detection algorithms.

## 1. Introduction

As one of the frontiers of current ocean development, deep-sea manned submersibles represent a country’s comprehensive scientific and technological strength in materials, control and marine disciplines [1]. As China’s first self-designed and self-developed operational deep-sea manned submersible, Jiaolong has performed many deep-sea dive missions and completed scientific investigations in the fields of marine geology, marine biology, and marine environment [2,3]. The fault detection of deep-sea manned submersibles has become one of the most significant tasks during the execution of the dive mission due to the person safety threat and economic loss caused by downtime of submersibles [4,5].

With the improvement of computing power and the development of signal processing technology, many researchers have made great achievements in the field of fault detection [6,7,8]. We can divide the fault detection methods into four categories: distance-based methods, clustering-based methods, probability distribution-based methods, and the deep learning-based methods. For distance-based methods, K-Nearest Neighbor (KNN) algorithm supposes that the k nearest neighbor distances of the fault sample are much larger than the normals’ [9]. However, KNN is suitable for the situations where the density of each cluster is relatively uniform. Local Outlier Factor (LOF) method pays more attention to the detection of local outliers, and the detected outliers can be considered as fault samples [10]. Density-Based Spatial Clustering of Applications with Noise (DBSCAN) [11], K-Means [12], and WaveCluster [13] are the representative algorithms of clustering-based methods. The limitation of them lies in requiring prior knowledge about data cluster number. In probability distribution-based methods, Gaussian Mixture Model (GMM) is a popular approach [14], which fits the dataset to a mixed Gaussian distribution, and discordant observations are probably caused by the failure events. However, the cluster type and number can act on the detection performance. In recent years, the deep learning-based methods have gained much popularity in fault detection [15,16,17,18,19]. In [20], a fuzzy neural network model combining BP neural network and fuzzy theory was established for fault diagnosis. A method based on a deep convolutional neural network was proposed for diagnosing bearing faults in [21]. Xu et al. proposed an fault diagnosis method based on deep transfer convolutional neural network [22], which combined transfer learning theory and convolutional neural network to realize online fault detection and diagnosis.

However, there are two critical problems in submersible fault detection.

(1) High-dimensional sensor data. The raw data from submersible sensing system is high in dimensionality, but redundant feature variables will bring challenges to fault detection and cause the increase in overfitting.

(2) Limited fault issues. Due to the low fault frequency of submersibles, only limited sensor data including fault samples is collected, which imposes limitations on model training and is a challenging problem for fault detection.

To address above-mentioned redundant features caused by high-dimensional dataset, a large collection of methods have been proposed, including Principal Component Analysis (PCA), Linear Discriminant Analysis (LDA) and feature selection composed of sub-approaches such as filter, wrapper, and embedded. In [23], PCA as an unsupervised dimensionality reduction method was used to remove redundant features in order to get the low-dimensional feature matrix and retain the essential attributes for the fault detection of rotor system. LDA processes labeled data, and when projecting them to a low-dimensional space, it satisfies as much as possible to retain the information of the data [24]. In [25], filter and wrapper methods were used to form a hybrid feature selection framework to get the best feature set, thereby improving the generalization and detection accuracy of model. In terms of small sample fault detection, data augmentation methods and siamese neural networks have become popular [26,27,28]. In order to obtain sufficient data and improve the robustness of the detection model, data augmentation is an significant technology in data processing [29]. Previously, methods such as noise addition, interpolation, window slicing, position replacement and sequence fusion have been maturely applied in data augmentation for fault detection [30]. With the development of deep learning, Generative Adversarial Networks (GAN) have been proposed as powerful tools for data generation [31]. In [32], a small-sample fault detection method using synthetic data was proposed, which improved GANs to generate more realistic fault data and enhance the detection accuracy. A multiple-objective generative adversarial active learning model was designed to detect outliers using limited data in high-dimensional space in [33]. In addition, siamese neural networks have made great achievements in small samples detection and one-shot learning [34], and to alleviate the over-fitting issue in anomaly detection of industrial cyber-physical systems, a siamese convolution neural network based few-shot learning model was proposed in [35].

We propose a novel high-dimensional and small-sample submersible fault detection method, which applies hierarchical clustering and AEs to select significant features and use GANs to synthesize data. In this paper, hierarchical clustering is used to cluster the raw data with the degree of similarity, and then AE is applied to evaluate the features of each cluster to determine the correlation between the feature groups and labels, so as to obtain the effective features for submersible fault detection. To get enough training data, a rough simulated data generation process is developed to transform the normal sensor data to rough simulated data according to adding adjusting rules in deep autoencoders. The improved DCGAN is the data refiner, which is trained to obtain realistic data transformed from the rough simulated data. Based on the above two processing methods, we have gotten a meaningful feature group and sufficient training data, so that CNN can be used for fault detection, which is pretrained and fine-tuned with generated data, and tested with the real sensor data.

The main contributions of this paper are as follows.

(1) A novel submersible fault detection method is designed, which innovatively completes the fault detection of submersible hydraulic system, and greatly improves the accuracy of detection by comparing with other classical algorithms.

(2) A feature selection model is proposed to select features strongly associated with fault event and able to effectively improve results of submersible fault detection and outperform several other state-of-art dimensional reduction methods.

(3) A data augmentation method based on improved DCGAN is developed, which generates more realistic data as training dataset for fault detection model. No real sensor data are required in fault detector training phase and the fault samples in submersible sensor dataset can be precisely detected.

The remainder of this paper is organized as follows. Section 2 is the description of the target submersible and sensor data set collected from the submersible. The submersible fault detection model is illustrated in Section 3. In Section 4, experiments and analysis are performed. Finally, Section 5 summarizes the conclusion of this paper with future work.

## 2. Data Description

The basic information of submersible and the data set used for fault detection are described in this section. Jiaolong is a manned submersible with a maximum working depth of 7000 m, and its detailed parameters are in Table 1. The data set contains a complete signal collected by the sensor system during a dive mission in the Southwest Indian Ocean hydrothermal area. The collection period spans about 11.5 h, and the sampling frequency is 2 Hz, resulting in a total of 83,856 observations.

The geological environment of the submarine is complicated and it is very prone to failure due to the existence of many “chimney”-like hydrothermal sulfides in the submarine hydrothermal area [36]. In this dive mission, the hydraulic system, load dumping and camera of the submersible failed, but only the failure of the hydraulic system was captured by the sensor signal. Furthermore, hydraulic system fault resulted in its corresponding multiple functions to fail, which greatly affected the operational capability of the submersible and brought a great threat to safety of the submersible.

**Figure 1:**
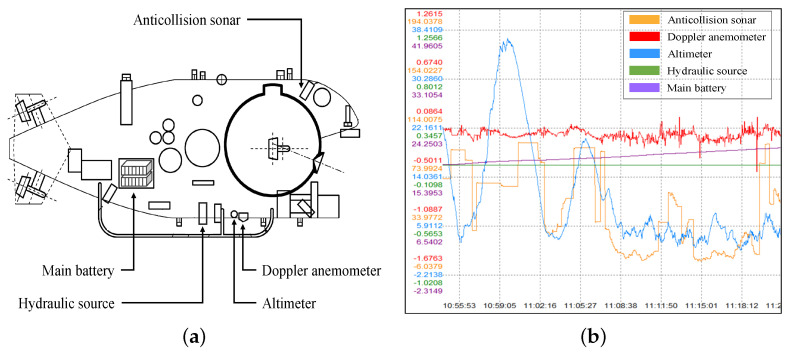
Structural sketch and corresponding sensing signals of Jiaolong submersible: (**a**) structural sketch of Jiaolong submersible; (**b**) sensing signals of Jiaolong submersible.

The raw data set consists of 294 features collected by sensors of multiple systems, which is related with hydraulic system, doppler anemometer, anticollision sonar, altimeter, main battery, etc. Figure 1 shows the some systems located in the submersible and their corresponding sensing signals. In this study, since the fault occurred in the hydraulic system, we only analyze the features related to the hydraulic system, and other features are not in our interest. The chosen 52 features are listed in Table 2.

## 3. Proposed Fault Detection Method

**Figure 2:**
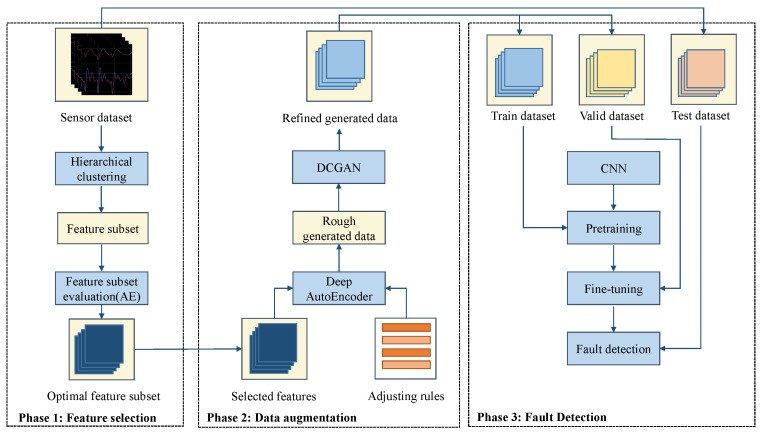
The overall architecture of the proposed fault detection method.

In this study, proposed feature selection module first extracts the essential features from the raw dataset. Then, DCGAN-based data augmentation module is proposed to generate sufficient training data. Finally, with the above-mentioned signal processing modules, CNN can achieve good performance under the challenges of high-dimensional data and limited fault data. The overall architecture of the fault detection method is shown in Figure 2.

### 3.1. Feature Selection

**Figure 3:**
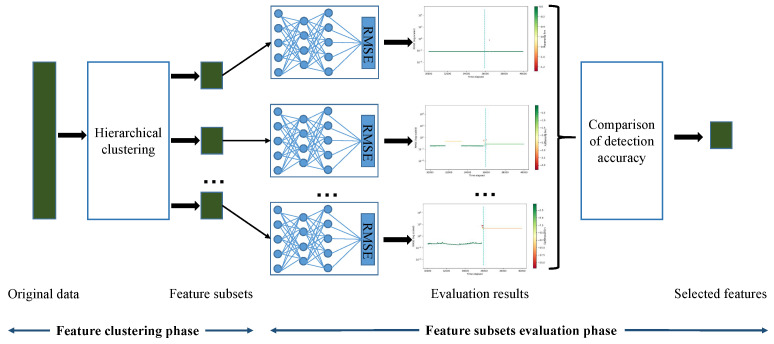
The architecture of feature selection module.

In this section, a novel feature selection module that is composed of a feature grouping method based on hierarchical clustering and AE-based feature evaluation is proposed, as shown in Figure 3.

#### 3.1.1. Features Clustering

**Figure 4:**
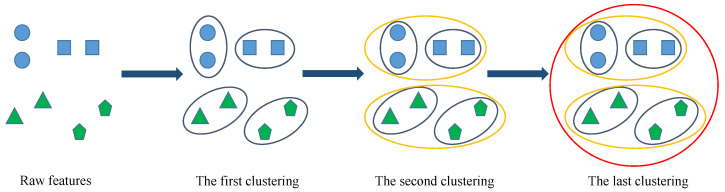
The sketch map of agglomerative hierarchical clustering algorithm.

In this work, agglomerative hierarchical clustering algorithm [37] is used as the feature grouping method. As shown in Figure 4, the agglomerative hierarchical clustering method initially treats each feature as a cluster, and then combines the two most similar clusters into a new larger cluster step by step. Iterate this process until all features are members of a single large cluster. In the clustering process, the correlation distance matrix is used to measure the similarity of two clusters, and then find clusters that can be further merged. The correlation distance $d_{c}$ between two feature variables $v_{i}$ and $v_{j}$ is expressed as follows.

(1) $$d_{c}\left(v_{i},v_{j}\right)=1-\frac{\left(v_{i}-\bar{v_{i}}\right)\cdot \left(v_{j}-\bar{v_{j}}\right)}{{∥v_{i}-\bar{v_{i}}∥}_{2}{∥v_{j}-\bar{v_{j}}∥}_{2}}$$

where $\bar{v_{i}}$ and $\bar{v_{j}}$ are the means of feature variables $v_{i}$ and $v_{j}$, respectively, and · represents the operation of dot product.

Let $N_{e}$ be the number of elements in each feature variable. Let $\vec{r}=\left\{r_{1},r_{2},\cdots ,r_{n}\right\}$, $r_{i}$, denoting the summed squared residuals of feature $v_{i}$, be defined as:

(2) $$r_{i}=\sum_{t=0}^{N_{e}}{\left(v_{i}^{\left(t\right)}-\bar{v_{i}}\right)}^{2}$$

The correlation distance matrix is denoted as *M*, and expressed as:

(3) $$M=\left[M_{i,j}\right]=1-\frac{C_{i,j}}{\sqrt{r_{i}}\sqrt{r_{j}}}$$

where

(4) $$C_{i,j}=\sum_{t=0}^{N_{e}}\left(\left(v_{i}^{\left(t\right)}-\bar{v_{i}}\right)\left(v_{j}^{\left(t\right)}-\bar{v_{j}}\right)\right)$$

$C_{i,j}$ represents the sum of residual products between features $v_{i}$ and $v_{j}$, and forms the partial correlation matrix $C_{r}$.

After obtaining the computing method of the correlation distance *M*, the agglomerative hierarchical clustering is carried out in the following steps.

(1) Apply *M* as the measure of the similarity between feature clusters, the clusters are gradually clustered into larger ones, and finally a large cluster containing all the features is obtained.

(2) Break the link of the current largest cluster and check whether the size of each cluster is less than δ.

(3) If the size of a cluster is greater than δ, repeat the processing in Step 2 until the size of all current clusters does not exceed δ.

(4) If the size of each cluster is less than δ, then current clusters are the feature groups that meet the requirements of clustering algorithm.

(5) *k* feature groups with strong correlation are obtained.

#### 3.1.2. Feature Subsets Evaluation

The feature variables in the raw data set are not all related to the fault, so it is very important to select the features that can help us detect the fault. On the premise that the features are divided into *k* feature subsets, in this section, the AE [38] is used to evaluate them and determine the feature groups that play a critical role in fault detection [39]. Let $G=\left\{g_{1},g_{2},\cdots ,g_{k}\right\}$, *G* is the set of *k* feature groups. As depicted in Figure 3, *k* three-layer AEs are applied to detect anomaly samples, which map to *k* feature subsets, respectively. As a anomaly detector, AE uses Root Mean Squard Error (RMSE) as the metric of anomaly score, and RMSE is defined as:

(5) $$\mathrm{RMSE}=\sqrt{\frac{1}{N_{e}}\sum_{i=1}^{N_{e}}{\left(x_{i}-x_{i}^{\prime }\right)}^{2}}$$

where $x_{i}$ and $x_{i}^{\prime }$ are the *i*th raw sample value and reconstruction value, respectively.

In the training phase, only normal data is used to train the AE model. Parameter set $\theta =\left\{\theta_{1},\theta_{2},\cdots ,\theta_{k}\right\}$, which is updated using Stochastic Gradient Descent (SGD). Furthermore, let $z_{train}=\left\{z_{1},z_{2},\cdots ,z_{k}\right\}$, where $z_{i}$ represents the RMSE vector of $g_{i}$ in training dataset. When predicting the anomaly value of the test data, the trained model is applied to get the RMSEs set $z_{predict}$ of test samples, which determines the anomaly property of each sample by setting the appropriate threshold μ. Finally, the optimal feature subset is selected by comparing threshold γ with the predicting accuracy. The training, predicting and evaluation phases of algorithm are presented in Algorithm 1.
**Algorithm 1** Feature subsets evaluation based on AE model.**Input**:G, set of feature groups.C, set of predicting sample labels.Nt, number of samples in training dataset.Np, number of samples in predicting dataset.μ, the RMSE threshold for anomaly samples.γ, the accuracy threshold for optimal feature subset.**Output**:
Gs, optimal feature subset.1:Initialize θ randomly;  //Training phase2:**for** $g_{i}\in G$ **do**3:  $z_{i}\leftarrow zeros\left(length=Nt\right)$;4:  **for** t←1..Nt **do**5:   $g_{i}^{\prime }\left[t\right]\leftarrow reconstruction\left(g_{i}\left[t\right],\theta_{i}\right)$;6:   $\theta_{i}$ in AE is updated;7:   $z_{i}\left[t\right]\leftarrow RMSE\left(g_{i}\left[t\right],g_{i}^{\prime }\left[t\right]\right)$;8:  **end for**9:**end for**10:$z_{train}\leftarrow \left\{z_{1},z_{2},\cdots ,z_{k}\right\}$, $\theta_{train}\leftarrow \left\{\theta_{1},\theta_{2},\cdots ,\theta_{k}\right\}$;  //Predicting phase11:**for** $g_{i}\in G$ **do**12:  $z_{i}^{\prime }\leftarrow zeros\left(length=Np\right)$;13:  **for** p←1..Np **do**14:   $g_{i}^{\prime }\left[p\right]\leftarrow reconstruction\left(g_{i}\left[p\right],\theta_{i}\right)$;15:   $z_{i}^{\prime }\left[p\right]\leftarrow RMSE\left(g_{i}\left[p\right],g_{i}^{\prime }\left[p\right]\right)$;16:  **end for**17:**end for**18:$z_{predict}\leftarrow \left\{z_{1}^{\prime },z_{2}^{\prime },\cdots ,z_{k}^{\prime }\right\}$;  //Evaluation phase19:**for** $g_{i}\in G$ **do**20:  **for** e←1..Np **do**21:   **if** $z_{i}^{\prime }\left[e\right]>\mu$ **then**22:    $Anomaly\left(g_{i}\left[e\right]\right)\leftarrow 1$;23:   **else**24:    $Anomaly\left(g_{i}\left[e\right]\right)\leftarrow 0$;25:   **end if**26:  **end for**27:  $L_{i}\leftarrow \left\{Anomaly\left(g_{i}\left[1\right]\right),Anomaly\left(g_{i}\left[2\right]\right),\cdots ,Anomaly\left(g_{i}\left[Np\right]\right)\right\}$;28:  Calculate $Accuracy(C_{i},L_{i})$;29:  **if** $Accuracy(C_{i},L_{i})>\gamma$ **then**30:   $Gs\left[i\right]\leftarrow g_{i}$;31:  **end if**32:**end for**

### 3.2. Data Augmentation

**Figure 5:**
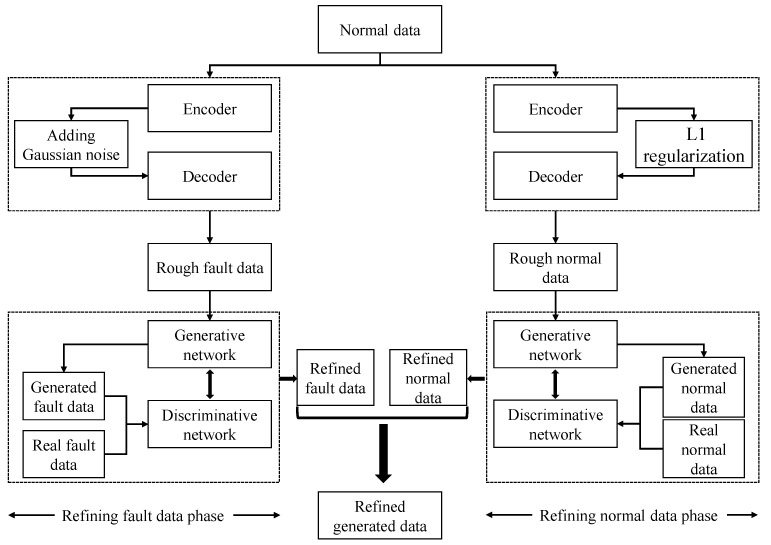
Flowchart of DCGAN-based data augmentation.

In order to prevent the scarce data set from reducing the effectiveness of fault detection and improve the generalization ability of detection model, data augmentation is a very important method to generate sufficient samples. The DCGAN-based data augmentation algorithm is proposed, where deep autoencoders are used in rough data generation and DCGANs are improved to refine rough generated data. Figure 5 shows the flowchart of the proposed data augmentation model.

#### 3.2.1. Rough Data Generation

**Figure 6:**
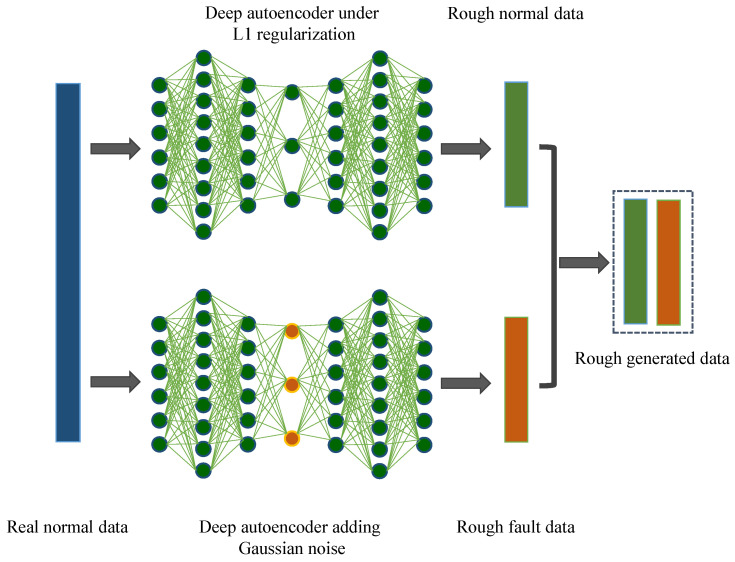
The process of generating rough data.

In this part, the method of generating rough data is illustrated in detail, where rough normal data is generated by encoder and decoder of deep autoencoder and the generation of rough data is guided by adjusting rules applied in deep autoencoder. The follow sections describe the detailed methods and the process of rough data generation is shown in Figure 6.

(1) Rough Normal Data Generation: The seven-layer AE subjecting to L1 regularization is used to produce the rough normal data, which is generated by first encoding and then decoding real normal data. Due to the same dimensions of input layer and output layer, the data converted by the deep autoencoder can be regarded as rough normal data.

(2) Rough Fault Data Generation: Contrast to the rough normal data generation process, the adjustment rules are applied to deep autoencoder in the simulated fault data generation, in which random Gaussian noise is added to the code layer to create rough fault samples deviating from normal ones.

#### 3.2.2. Generated Data Refining

The rough generated data cannot be directly applied to the submersible fault detection, as there is still a big gap between it and real data. In this part, the improved DCGAN is used as the rough data refiner.

**Figure 7:**
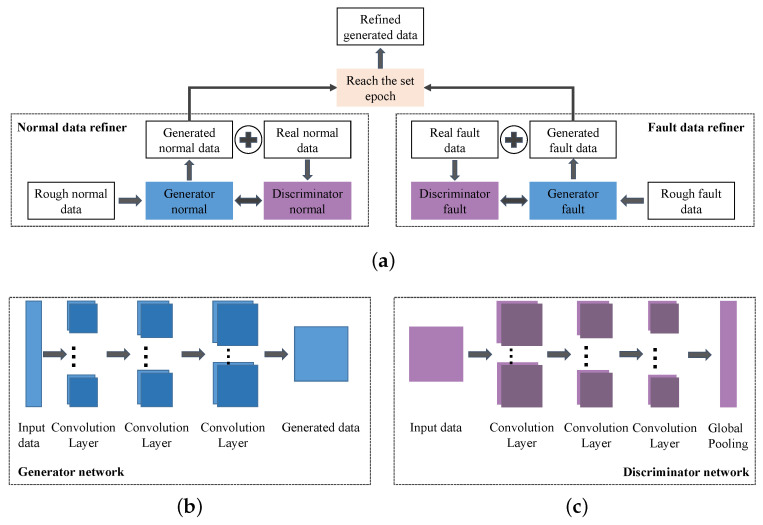
The architecture of data refiner: (**a**) the basic architecture of DCGAN-based normal data refiner and fault data refiner; (**b**) structure of generator networks; (**c**) structure of discriminator networks.

(1) Deep Convolutional Generative Adversarial Network: DCGAN is an unsupervised learning algorithm that combines CNN and GAN [40]. As shown in Figure 7a, similar to the general GAN, it consists of a generator *G* and discriminator *D*, and can be described in the following equation:

(6) $$V\left(G,D\right)=E_{x∼P_{data}}\left[logD\left(x\right)\right]+E_{z∼P_{z}}\left[log\left(1-D\left(G\left(z\right)\right)\right)\right]$$

where *x* and *z* are the real data with the distribution $P_{data}$ and data as the input of *G* with the distribution $P_{z}$, respectively. Function *D*() represents the probability that the discriminated data is from real data and the optimum GAN is expressed as the follow equation.

(7) $${\mathrm{GAN}}^{*}=arg\;\min_{G}\;\max_{D}V\left(G,D\right)$$

where *D* is trained to maximize $D\left(x\right)$ and $1-D\left(G\left(z\right)\right)$ so as to correctly identify real data and generated data, and *G* is trained to minimize $1-D\left(G\left(z\right)\right)$, so that generated data is more realistic.

(2) Rough Data Refiner Based on DCGAN: In order to better combine CNN and GAN, DCGAN replaces pooling operation with strided convolution in both generating network and discriminating network, and uses global pooling layer instead of fully connected layer to improve model stability. The detailed structures of generator networks and discriminator networks are shown in Figure 7b,c, respectively. Then, the generator loss $L\left(G\right)$ and discriminator loss $L\left(D\right)$ are calculated in Equations (8) and (9).

(8) $$L\left(G\right)=\frac{1}{N}\sum_{i=1}^{N}-log\left(D\left(G\left(z_{i}\right)\right)\right)$$

(9) $$L\left(D\right)=\frac{1}{N}\sum_{i=1}^{N}-log\left(D\left(x_{i}\right)\right)-log\left(1-D\left(G\left(z_{i}\right)\right)\right)$$

To make the generated data closer to real data, loss function of generator is improved according to actual submersible sensor conditions and is described as follows:

(10) $$L_{improved}\left(G\right)=L\left(G\right)+L_{similarity}\left(G\right)=\frac{1}{N}\sum_{i=1}^{N}\left[-log\left(D\left(G\left(z_{i}\right)\right)\right)+\lambda \left(1-\frac{G\left(z_{i}\right)\cdot x_{i}}{∥G\left(z_{i}\right)∥∥x_{i}∥}\right)\right]$$

where $L_{similarity}$ is the loss from cosine similarity between generated data and real data and λ is the weight of cosine similarity loss.

Based on the above loss functions, the parameters of generating networks and discriminating networks are updated by SGD in the training process. As shown in Figure 7a, rough normal data and fault data are, respectively, processed by refiners composed of generators and discriminators, and when training epochs reach 200, the data from generators can be determined to be refined generated data.

### 3.3. Fault Detection Based on CNN

#### 3.3.1. Data Preprocessing

In general, the data collected from sensor in the submersible is one-dimensional waveform signal data, but it can also be presented as two-dimensional grayscale images. In this work, a sensor data preprocessing method is proposed to convert waveform signal data to image data which are the ideal inputs for CNN model.

**Figure 8:**
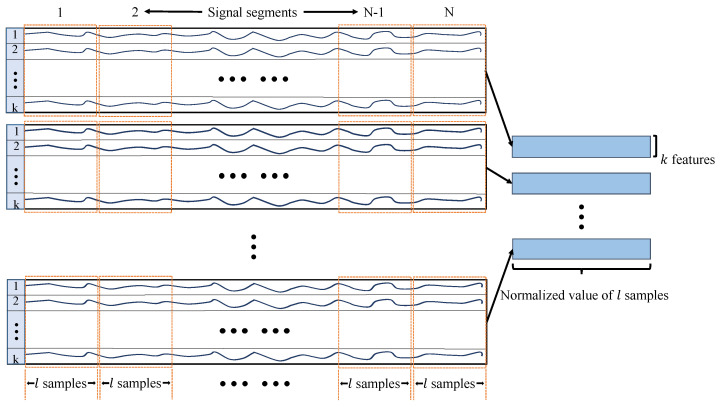
Proposed sensor data processing method.

The detailed process of data preprocessing method is shown in Figure 8. Let signal data including *k* feature variables be evenly divided into *N* parts, and each segment have *l* samples. Since the signal data has a lower dimensionality compared with general images, so the method that $\frac{l}{k}$ data segments are repeatedly used to form a l×l matrix. To transform the matrix to a grayscale image, the value of each element in matrix is normalized from 0 to 255 and then used as gray level of a pixel in the image. The normalization method is designed as following:

(11) $$Gray\left(i,j\right)=int\left(255\times \frac{Matrix\left(i,j\right)-\min\left(Matrix\right)}{\max\left(Matrix\right)-\min\left(Matrix\right)}\right)$$

where Gray() and Matrix() are the value of pixel in grayscale image and the element value in matrix, respectively, and function int() makes fractions round down. The preprocessing algorithm does not require the guidance of prior knowledge and the obtained images can maintain the characteristics of raw data as much as possible.

#### 3.3.2. Proposed Fault Detection Framework

In this section, a submersible fault detection framework based on CNN model with LeNet-5 structure is described. As shown in Figure 2, the framework consists of three parts: pretraining and fine-tuning using synthetic dataset, and fault detection testing using test set in real data.

**Figure 9:**
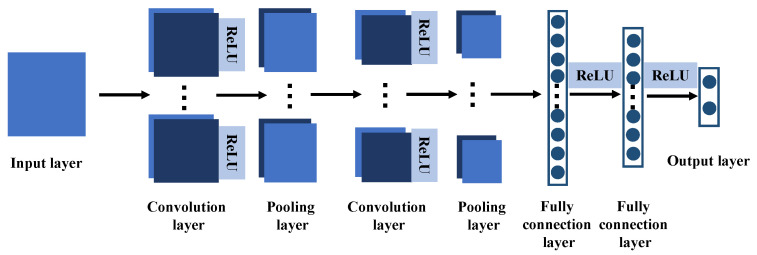
The network structure of LeNet-5.

The LeNet-5 was originally proposed as convolutional neural network model for handwritten digit recognition and had achieved good results in image recognition and classification [41,42]. The structure of LeNet-5 is shown in Figure 9, in which there are one input layer, two convolution layers, two pooling layers, two fully connection layers and one output layer. ReLU function follows each convolution layer as an activation function and provides the sparse representation ability of the neural network.

In view of the fact that only one fault issue in the hydraulic system, the data used for submersible fault detection is very limited. In addition, if the detection model is directly trained with real data, it will increase the risk of overfitting, and cannot prove the effectiveness of our model. so we apply synthetic data to pretraining and fine-tuning of fault detection model. After obtaining the pretrained model, only a small portion of the generated data is used to fine-tune parameters of fully connection layers to get the model applied to real data. Based on the established framework and the trained model, the real sensor data can be detected whether a fault has occurred.

## 4. Experimental Result

### 4.1. Experiment Settings and Results

Feature selection, data augmentation and fault detection are the three parts of the proposed method. This section illustrates experiment settings and results of each part. All the numerical experiments are carried out with Python 3.5 and run on workstation equipped with an Intel 3.80 GHz CPU, RTX3060 GPU and 16.0-GB RAM.

#### 4.1.1. Feature Selection Experiment

In feature selection section, normal samples are used for hierarchical clustering and the training of AEs in feature subsets evaluation and samples are used as predicting dataset for AEs, half of which are from normal dataset and the other half are part of fault samples.

**Figure 10:**
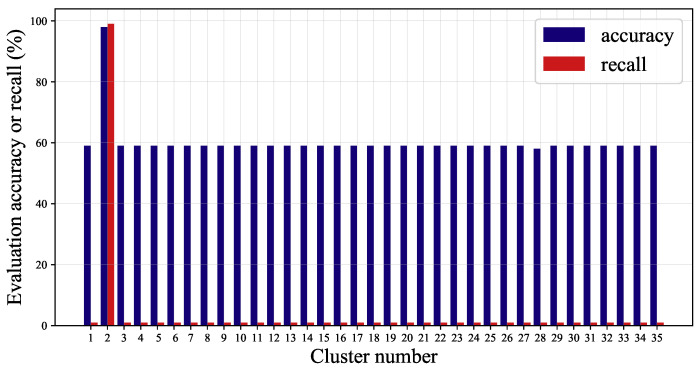
The evaluation results of feature subsets.

The maximum size δ of cluster is set to 1 in feature clustering and the clustering results are listed in Table 3. The thresholds in feature subsets evaluation are set as: μ=1, γ=0.95, and the accuracy and recall rate of each feature subset obtained by evaluation are shown in Figure 10. According to the evaluation results, we can find that accuracy and recall rate of Cluster 2 have reached 0.98 and 0.99, respectively, while the evaluation results of other clusters are very poor, indicating that only features in Cluster 2 have strong correlation with fault event and others are not helpful for fault detection.

#### 4.1.2. Data Augmentation Experiment

In this part, 30,000 signal samples have been selected from normal dataset to generate rough data, including 15,000 rough normal signal samples and 15,000 rough fault signal samples, respectively, which are preprocessed into 32×32 image data for subsequent data refining. In addition, setting the preprocessed image sample size to 32×32 takes into account the time required for the occurrence of submersible failure (approximately 10 to 20 s) and the sample size is set to an exponential power of 2 to facilitate the calculation of CNN. DCGAN is used as a refiner and the network structures of its generator and discriminator are listed in Table 4. Normal data refiner and fault data refiner share the same network structure in generators and discriminators, but the parameters of them are individually trained. Here, Convolution 1 (128@4×4) indicates that there are 128 convolutional kernel of size 4×4 in this layer. The initial settings of hyperparameters for all networks are 0.0002 for learning rate, 64 for mini-batch size and 300 for max-epoch.

**Figure 11:**
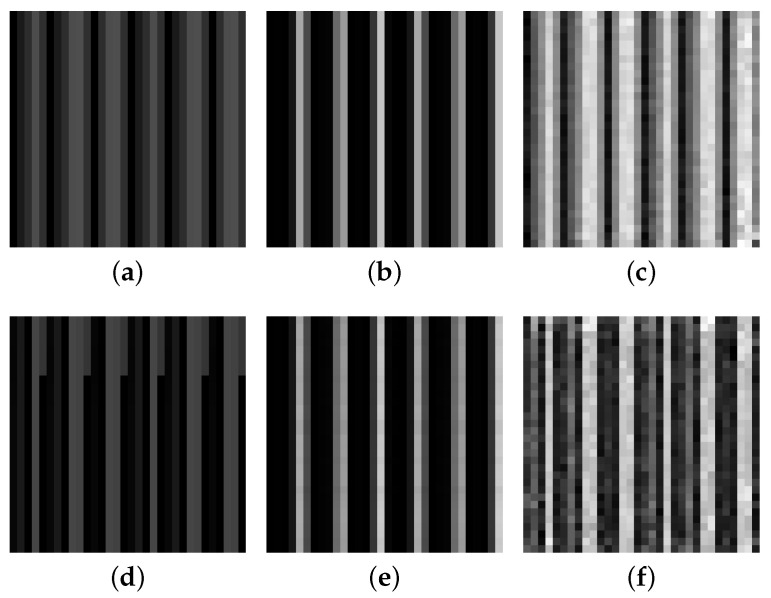
Results of data generation. The first row of data is the normal data, whereas the second row is the fault data: (**a**) real normal data; (**b**) rough normal data; (**c**) refined normal data; (**d**) real fault data; (**e**) rough fault data; (**f**) refined fault data.

Using the proposed generating data model, a large amount of realistic normal data and fault data can be created and Figure 11 illustrates the real data, rough data and refined data. In reality, there is a difference between normal data and fault data in numerical values and changing trends, but after signal data is converted to grayscale images, the difference seems to be small to us, however, classifiers can clearly distinguish them. As shown in Figure 11, the refined data is closer to the real data than the rough data, and it is able to show the different characteristics of normal data and fault data.

**Figure 12:**
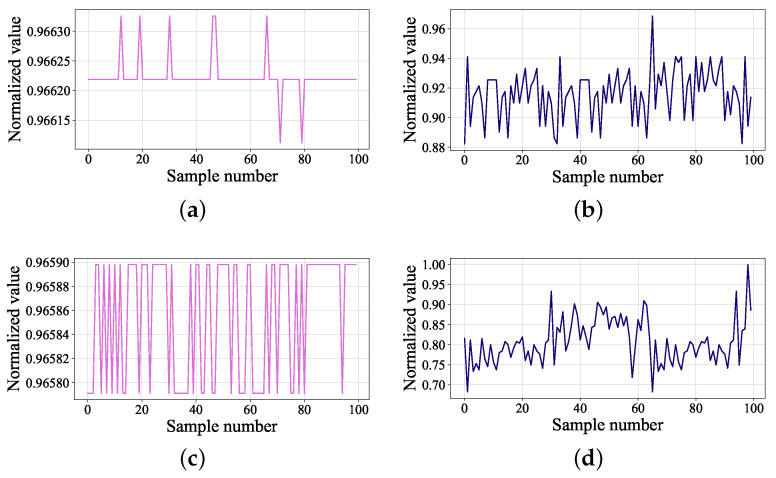
Real data and generated data: (**a**) real normal data of temperature of tank VP2; (**b**) generated normal data of temperature of tank VP2; (**c**) real fault data of temperature of tank VP2; (**d**) generated fault data of temperature of tank VP2.

In order to compare the real data and generated data more clearly, the details of real data and synthetic data of temperature of tank VP2 under normal and anomaly conditions are shown in Figure 12. Obviously, the value of fault data is lower than normal data and has been in a fluctuating state, while the normal data occasionally changes in value. In terms of numerical value, generated fault data is also smaller than generated normal data. Since the generated data contains more noise, the generalization of the classifier can be improved and overfitting can be prevented.

#### 4.1.3. Fault Detection Experiment

**Figure 13:**
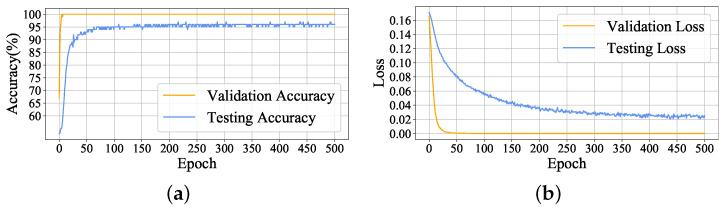
Fault detection experiment result: (**a**) validation accuracy and testing accuracy; (**b**) validation loss and testing loss.

In fault detection phase, 2448 image samples are generated from DCGAN, 80% of which are applied in pretraining phase and the remaining data is used in fine-tuning phase. Real data set is divided into 2994 images, and all of them are testing samples. Here, both synthetic data and real data are preprocessed into 32×32 images. The structure of LeNet-5 is detailed in Table 5, and the initial parameters of model are set as follows: learning rate is 0.0001, the min-batch size is set as 4 and max-epoch is 500. In this case, the model is first trained by synthetic data, and then, the fully connection layers are fine-tuned by the valid dataset from generated data. Finally, the trained model can carry out fault detection on the test dataset. The accuracy and the values of loss function of proposed method in validation and testing process are shown in Figure 13. At the validation stage, since model is still detecting synthetic data, the accuracy quickly reaches 100%. Moreover, it can be seen that a stable testing accuracy of 97% has been achieved and loss function gradually converges during the testing stage.

### 4.2. Comparative Experiments and Analysis

In this section, the proposed submersible fault detection method is evaluated by three comparative experiments. Comparative experiments in Section 4.2.1 are designed to verify that the proposed feature selection method is able to improve the accuracy of fault detection and outperforms other dimensionality reduction methods. In Section 4.2.2, experiments are conducted to examine the effect of different numbers of generated training samples on the submersible fault detection. Finally, experiments comparing our proposed method with three classic fault detection algorithms are carried out to verify the superiority of proposed method in Section 4.2.3.

#### 4.2.1. Comparative Experiments with Different Feature Selection Methods

Four groups of fault detection experiments are carried out. In Group 1, feature selection process is not performed before fault detection, whereas PCA, Recursive Feature Elimination (RFE) and our proposed feature selection method are applied to Group 2, Group 3 and Group 4, respectively. Three general fault detection methods (LOF, isolation forest and one-class SVM) are used in the comparative experiments.

**Figure 14:**
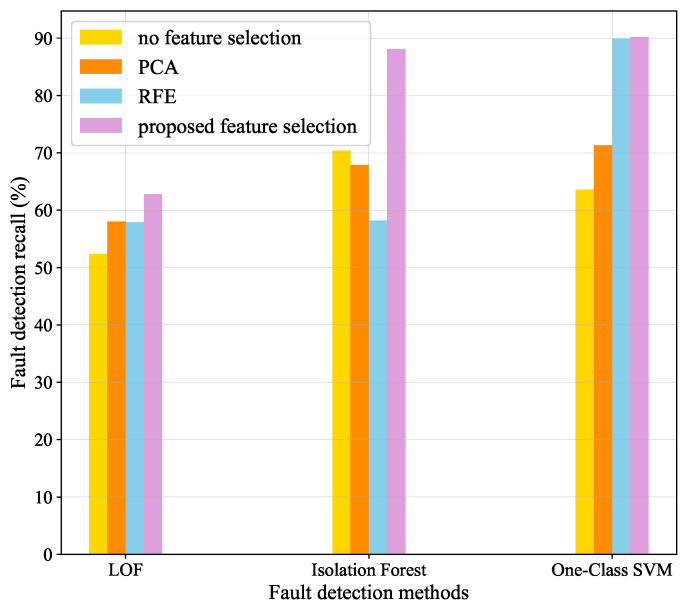
Comparison results of three fault detection methods with three feature selection algorithms.

Figure 14 shows the results of four groups experiments. With the different feature selection methods, the following results hold:

(1) When using LOF method, our proposed method has made the greatest contribution to improving detection accuracy, whereas PCA and RFE can only improve fault detection slightly.

(2) When using isolation forest method, only our method can greatly improve the performance of fault detection, and other methods will reduce the accuracy of detection.

(3) When using one-class SVM method, both RFE and our method can greatly improve the accuracy of fault detection and our method outperforms RFE by 0.3%. However, the improvement effect of PCA is relatively small.

In short, after processing of the proposed feature selection method, the accuracy of three fault detection method has been greatly improved, and our method outperforms PCA and RFE.

#### 4.2.2. Comparative Experiments with Different Numbers of Generated Samples

**Figure 15:**
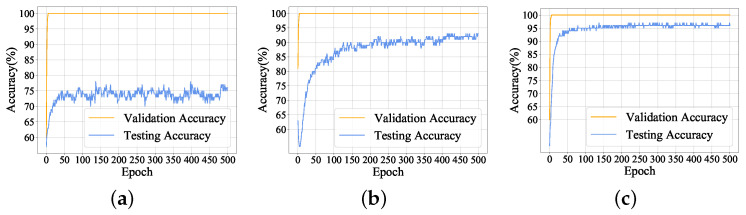
Comparison results of validation accuracy and testing accuracy with different numbers of training samples: (**a**) 1000 training samples; (**b**) 1400 training samples; (**c**) 2000 traning samples.

In this section, a comparative experiment is given to illustrate that the number of generated samples for training LeNet-5 model can affect the detection accuracy of fault detection model. The structure and parameters of detection model, validation dataset and testing dataset remain the same, whereas the number of training samples changes. We set the number of training samples to 1000, 1400 and 2000, then train, fine-tune and test the detection model, respectively. As shown in Figure 15, although the validation accuracy in the three experiments can reach 100%, the test accuracy improves as training samples increase. When the number of training samples is 1000, testing accuracy cannot reach 85%, whereas the testing accuracy is able to go up to 97% as the samples number increases to 2000. It can be concluded that the number of training samples has a great influence on the performance of fault detection.

#### 4.2.3. Comparative Experiments with Classic Fault Detection Algorithms

In order to verify the effectiveness and superiority of the proposed method, the performances of classic fault detection methods including isolation forest, LOF and one-class SVM are given for comparison. As shown in Table 6, accuracy, recall, precision and F1 are used as metrics to compare the performance of fault detection. We observe that our proposed method has achieved the best results in accuracy, recall, precision and F1 at 0.97, 0.98, 0.96 and 0.97, respectively, and they are significantly better than other methods.

### 4.3. Failure Analysis of Submersible Hydraulic System

**Figure 16:**
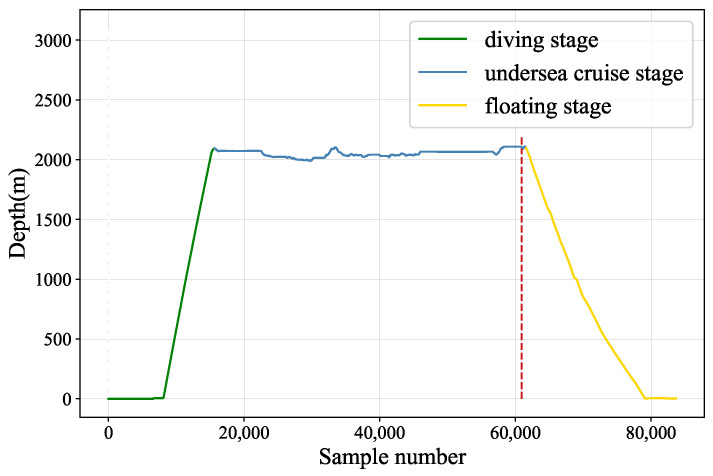
Depth values during the dive.

**Figure 17:**
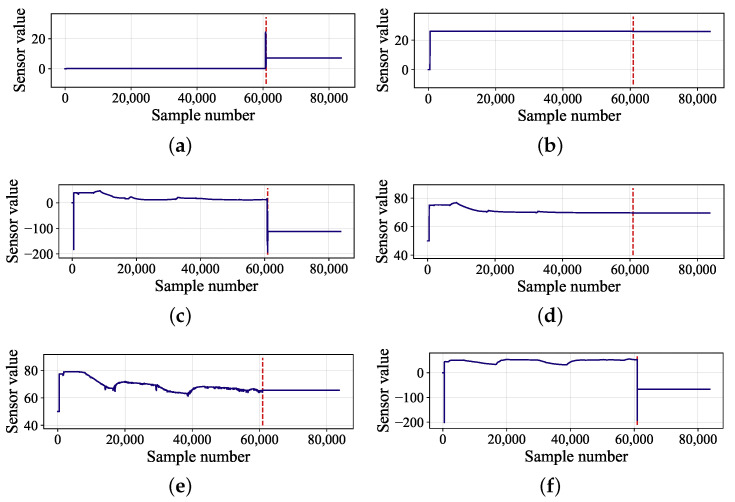
Sensor variables related to hydraulic system fault event: (**a**) current of 24V power; (**b**) tank pressure; (**c**) temperature of tank VP2; (**d**) displacement of compensator 10LPM; (**e**) displacement of compensator 15LPM; (**f**) temperature of tank VP1.

We conduct analyse to find the relationship between the failure of hydraulic system and the related sensor variables. In this dive mission, hydraulic oil leaked into the main valve box due to solenoid valve leakage at a depth of 2100 m. The liquid in the valve box with limited volume continued to accumulate, causing the pressure to rise, and the pressure was acting on the valve plate. The valve plate burst and sea water entered the valve box. The Electronic Control Unit (ECU) board in the valve box was short-circuited and burned in contact with water, and the current suddenly changed, which exceeded the bearing range of the relay after the air switch of the hydraulic system in the cabin, and the relay was burned out. Subsequently, the values of various sensors in the hydraulic system failed. Figure 16 shows the depth variation information during the dive of submersible and the six subgraphs in Figure 17 represent six sensor variables, where the red dashed lines mark the points of failure.

As shown in Figure 17a, since the valve plate of the hydraulic valve box burst and sea water entered the valve box, which caused the ECU board to short-circuit and burn, so the current from 24V power supply suddenly changed drastically. Moreover, activating signals of main hydraulic source and auxiliary hydraulic source were located on the ECU control board of main valve box, so that the failure caused the entire hydraulic system to be paralyzed. Therefore, the signals from tank pressure (see Figure 17b), temperature of tank VP1 (see Figure 17f) and displacement of compensator 15LPM (see Figure 17e) located in main valve box and temperature of tank VP2 (see Figure 17c) and displacement of compensator 10LPM (see Figure 17d) located in auxiliary valve box did not work properly.

By analyzing the fault events and sensor signals, we further understand the specific details of the fault and also discover the design loopholes in hydraulic system of the submersible. In the follow-up study, experts improve the hydraulic system to separate the activating signals of main and auxiliary hydraulic sources, so as to avoid similar incidents in the future.

## 5. Conclusions

In this paper, a submersible fault detection method is proposed. The method is designed to overcome the difficulties of scarce dive data and high dimensionality. There are three modules in this method: feature selection, data augmentation and fault detection. In the first module, agglomerative hierarchical clustering and AEs are used to select the optimal feature subset related to the fault event. In the second module, the proposed adjusting rules is used to generated rough data with deep autoencoders, then the improved DCGAN as refiner transforms the rough data to realistic data. In the third module, LeNet-5 structure-based CNN model is applied as the fault detector, which is trained and fine-tuned with generated data. The proposed method is tested by the real submersible sensor data, and the results indicate that our method can effectively detect fault occurring in submersible hydraulic system. In comparison with several classic algorithm, in terms of accuracy, recall, precision and F1, the proposed method outperforms other fault detection algorithms. We have also analyzed the relationship between fault event and sensor signals, which can provide information for the retrospect of the fault details.

Although good results have achieved in this paper, there are still some limitations in our study. First, we currently only detect and analyze the failure of the hydraulic system in the submersible. Second, our proposed method currently only processes the sensor signal of the submersible. Third, we can only simulate the fault occurred to generate data. Therefore, we will continue the study focusing on three aspects: (1) After obtaining the fault data of other systems, we will improve the algorithm according to its data characteristics to achieve accurate fault detection. (2) The adaptive improvement of the algorithm is made to transfer it to other data sets so as to realize the fault detection of other applications. (3) We will introduce expert knowledge in fault data generation to detect possible faults.

## Figures and Tables

**Table 1:** Parameters of the Jiaolong manned submersible.

Parameters	
Length	8.6 m
Breadth	3.9 m
Height	3.4 m
Weight in air	22.3 t
The inner diameter of the manned spherical shell	3.4 m

**Table 2:** The features of hydraulic system.

Feature Name	Description
Pressure of system [VP1, VP2]	The pressure values of main hydraulic system and auxiliary hydraulic system
Current of [110V power, 24V power]	The current values of main power and auxiliary power
Tank pressure	The pressure values of fuel tank
Temperature of tank [VP1, VP2]	The temperature of main fuel tank and auxiliary fuel tank
Displacement of compensator [10LPM, 15LPM]	Displacement values of main compensator and auxiliary compensator
Trim system level compensation alarm	Alarm conditions of liquid level compensation in trim system
Leak	Leakage of hydraulic system
Backup [1, B1, A5, B5, A12, B12]	Six types of backup data
Microbial sampler	Working conditions of the microbial sampler
Submerged drilling work [A2, B2]	Working conditions of the two submersible drills
Trim pump power [A3, B3]	Power of two trim pumps
Abandonment of main manipulator [A4, B4]	Abandonment conditions of two main manipulators
Main manipulator work [A6, B6]	Working conditions of two main manipulators
Deputy manipulator work [A7, B7]	Working conditions of two deputy manipulator
Conduit pulp rotary mechanism [A8, B8]	Two types of conduit pulp rotary mechanism
Load of [VP1, VP2]	Load of main hydraulic system and auxiliary hydraulic system
Sea water pump signal	Signal from sea water pump
Control signal of [15LPM, 10LPM, 1.2LPM]	Three types of control signal
Sea valve [A9, B9, A10, B10, A11, B11]	Six types of sea valve signal
Floating load rejection A13	Load rejection conditions in floating
Diving load rejection B13	Load rejection conditions in diving
Abandonment of deputy manipulator [A14, B14]	Two types of abandonment of deputy manipulator
Ballast tank drainage [A15, B15]	Two types of drainage ballast tank
Ballast tank inflow [A16, B16]	Two types of inflow ballast tank
Proportional valve adjusts the trim angle [1, 2]	Two trim angles in proportional valve adjusting

**Table 3:** Feature clustering results.

Clusters	Features
Cluster 1	Main manipulator work A6
	Current of 110V power
	15LPM control signal
	Pressure of system VP1
	VP1 load
Cluster 2	Temperature of tank [VP1, VP2]
	Current of 24V power
	Tank pressure
	Displacement of compensator [10LPM, 15LPM]
Cluster 3	Sea water pump signal
	Sea valve [BC A10, BC B10, AD B9]
	Backup B12
	Ballast tank inflow A16
	Pressure of system VP2
	10LPM control signal
	VP2 load
Cluster 4∼35	Each of the remaining 32 features is a cluster

**Table 4:** Structures of generators and discriminators.

Layers in Generators	Layers in Discriminators
Input (100×1)	Input (32×32)
Convolution 1 (128@4×4)	Convolution 1 (32@16×16)
Convolution 2 (64@8×8)	Convolution 2 (64@8×8)
Convolution 3 (32@16×16)	Convolution 3 (128@4×4)
Output (32×32)	Global pooling (128×1)
	Output ($D\left(x\right)$)

**Table 5:** Structure of LeNet-5 model.

Layers in LeNet-5
Input (32×32)
Convolution 1 (16@28×28)
Pooling 1 (2×2)
Convolution 2 (32@10×10)
Pooling 2 (2×2)
Fully connection 1 (120)
Fully connection 1 (84)
Output (2)

**Table 6:** Fault detection performance comparisons.

Methods	Accuracy	Recall	Precision	F1
Proposed method	0.97	0.98	0.96	0.97
Isolation forest	0.70	0.87	0.75	0.81
LOF	0.52	0.72	0.66	0.69
One-class SVM	0.64	0.76	0.89	0.82

## Data Availability

Not applicable.
